# Genome-Wide SNP Discovery, Genotyping and Their Preliminary Applications for Population Genetic Inference in Spotted Sea Bass (*Lateolabrax maculatus*)

**DOI:** 10.1371/journal.pone.0157809

**Published:** 2016-06-23

**Authors:** Juan Wang, Dong-Xiu Xue, Bai-Dong Zhang, Yu-Long Li, Bing-Jian Liu, Jin-Xian Liu

**Affiliations:** 1 Key Laboratory of Marine Ecology and Environmental Sciences, Institute of Oceanology, Chinese Academy of Sciences, Qingdao, Shandong, China; 2 Laboratory for Marine Ecology and Environmental Science, Qingdao National Laboratory for Marine Science and Technology, Qingdao, China; 3 University of Chinese Academy of Sciences, Beijing, China; Chinese Academy of Fishery Sciences, CHINA

## Abstract

Next-generation sequencing and the collection of genome-wide single-nucleotide polymorphisms (SNPs) allow identifying fine-scale population genetic structure and genomic regions under selection. The spotted sea bass (*Lateolabrax maculatus*) is a non-model species of ecological and commercial importance and widely distributed in northwestern Pacific. A total of 22 648 SNPs was discovered across the genome of *L*. *maculatus* by paired-end sequencing of restriction-site associated DNA (RAD-PE) for 30 individuals from two populations. The nucleotide diversity (π) for each population was 0.0028±0.0001 in Dandong and 0.0018±0.0001 in Beihai, respectively. Shallow but significant genetic differentiation was detected between the two populations analyzed by using both the whole data set (*F*_ST_ = 0.0550, *P* < 0.001) and the putatively neutral SNPs (*F*_ST_ = 0.0347, *P* < 0.001). However, the two populations were highly differentiated based on the putatively adaptive SNPs (*F*_ST_ = 0.6929, *P* < 0.001). Moreover, a total of 356 SNPs representing 298 unique loci were detected as outliers putatively under divergent selection by *F*_ST_-based outlier tests as implemented in BAYESCAN and LOSITAN. Functional annotation of the contigs containing putatively adaptive SNPs yielded hits for 22 of 55 (40%) significant BLASTX matches. Candidate genes for local selection constituted a wide array of functions, including binding, catalytic and metabolic activities, etc. The analyses with the SNPs developed in the present study highlighted the importance of genome-wide genetic variation for inference of population structure and local adaptation in *L*. *maculatus*.

## Introduction

Considering the ongoing worldwide depletion of most marine populations [1], accurate estimates of population demographic parameters are often necessary for fisheries management [2, 3]. In the past decades, tens to hundreds of neutral markers have been used for population genetic inference [4–6]. However, the applications for recently isolated populations of marine species with shallow genetic structure and large effective population size have been limited.

Genome-wide genetic variations can provide reliable estimates of population demographic parameters [7–9] and identify genomic regions under selection [10–12]. Genome-wide SNPs have been successfully used to elucidate population structure of marine fishes including Pacific lamprey (*Entosphenus tridentatus* [13]), Atlantic salmon (*Salmo salar* [14]) and European eel (*Anguilla anguilla* [15]). Moreover, studies based on genome scan have also discovered adaptively important candidate genes and genomic regions in non-model fish species including three-spined stickleback (*Gasterosteus aculeatus* [16]), Sockeye salmon (*Oncorhynchus nerka* [17]), Chinook salmon (*Oncorhynchus tshawytscha* [18]), Atlantic cod (*Gadus morhua* [19]) and turbot (*Scophthalmus maximus* [20]).

In recent years, advances in high-throughput reduced-representation genome sequencing (RRGS) technology have provided an unprecedented opportunity to conduct population genomic studies in both model and non-model organisms. Restriction-site associated DNA tag sequencing (RAD-seq) is a powerful RRGS protocol [21, 22]. RAD-seq approach has been successfully applied in a variety of organisms to identify resources of genome-wide SNPs, including both plants [23, 24] and animals [25, 26]. The advantages of RAD-seq in efficiency, costs and accuracy have revolutionized the field of population genetics and facilitated population structure inferences and local adaptation studies at a genome wide scale [27].

The spotted sea bass, *Lateolabrax maculatus*, belongs to the family Moronidae (Perciformes) [28, 29]. *Lateolabrax maculatus* is distinguished newly described species from the Japanese sea bass, *L*. *japonicus* and is characterized by many clear black dots on lateral body region [30]. It is widely distributed along coasts of the Bohai Sea, Yellow Sea, East China Sea and South China Sea, reaching south to borders between China and Vietnam and north to Southeast coast of South Korea [31, 32]. *L*. *maculatus* is a species of high commercial value and mainly found in moving water of inshore rocky reefs. Population decline of *L*. *maculatus* has been recorded due to overfishing and habitat deterioration resulting from anthropogenic activities [33, 34]. Although previous population genetics studies using both mitochondrial DNA (mtDNA) sequences and microsatellites showed some genetic structuring between populations of *L*. *maculatus* [32, 33], fine-scale population structure still remains to be revealed by genomic-wide genetic data. Moreover, the Northwest Pacific marginal seas provide an excellent natural system for studying local adaptation. The Northwest Pacific marginal seas are relatively young postglacial ecosystems (< 10 000 years) and characterized by environmental gradients [32]. For example, the average annual sea surface temperature ranged from 10.9°C in Bohai Sea to 26.5°C in South China Sea (data provided by the National Oceanic and Atmospheric Administration; NOAA). As a widely distributed marine fish species in the Northwest Pacific, populations of *L*. *maculatus* may experience divergent selection in heterogenous environments. Furthermore, naturally spawned fry of *L*. *maculatus* were captured from coasts of China, Korea, and Taiwan and transported to different regions of China, Japan and Korea for cage cultivation in the past three decades [35, 36]. The development of a set of appropriate molecular markers will also facilitate the scientific management of the genetic resource and the avoidance of the genetic disturbance of the natural populations caused by the occasional escape of cultured individuals.

In the present study, we generated a novel resource of genome-wide SNPs for *L*. *maculatus* by paired-end sequencing of restriction-site associated DNA (RAD-PE) for 30 individuals collected from two populations across its distribution range in China. The SNPs were then used to evaluate the levels of genetic diversity and population divergence between the two populations. Outlier tests were also conducted to detect loci under putative selection. Finally, function annotation of the outlier loci was performed to determine whether the potentially adaptive loci localized to known genes or conserved genomic regions.

## Materials and Methods

### Ethics statement

The field studies did not involve any endangered or protected species. *Lateolabrax maculatus* is not protected by Chinese law. No fishing license was required for collection of samples from all locations. It is a commercially harvested species in China. The fish were collected by trawling by local fishermen for commercial purposes and were already dead when collected. No of the authors was involved in the collection of the fish. Animal Ethics Committee approval was not needed because no handing of live animals was involved.

### Sample collections and DNA extraction

Samples were collected from two separate locations of heterogenous environments in May 2014: one from coast of Beihai, Guangxi Province (21°24’ N, 109°05’ E, *T*_a_ = 26.5°C, *T*_a_, average annual sea surface temperature) and the other from Dandong, Liaoning Province (39°52’ N, 124°19’ E, *T*_a_ = 10.9°C). Muscle tissue samples of a total of 30 individuals (16 from Beihai and 14 from Dandong) were collected and preserved in 96% ethanol for DNA extraction. Genomic DNA was extracted from ~100 mg muscle tissue using a standard phenol-chloroform extraction protocol [37]. Samples were treated with RNase A to produce pure, high molecular weight, RNA-free DNA. Quality and concentration of DNA samples were measured by a Nanodrop^TM^ 2000 (Thermo Scientific) spectrophotometer and a Qubit^®^2.0 fluorometric quantitation. The optimal concentration was no less than 50 ng/μL, and the total DNA recovered was more than 2 μg.

### Library preparation and sequencing

RAD-PE libraries were prepared using the protocol outlined by Baird *et al*. [21] and Etter *et al*. [38]. Genomic DNA from each individual was digested with high fidelity restriction enzyme *Eco*RI (G^AATTC). Then, Illumina P1 adapter containing individual-specific index (6 bp) was ligated to the digested products. The adapter-ligated DNA was sheared and separated by electrophoresis on a 2% agarose gel. Fragments in the 200–600 bp size range were collected using a MinElute Gel Extraction Kit (QIAGEN, Beijing). After treating double-stranded DNA ends with blunt-ending enzymes and adding 3’-adenine over-hangs, a modified Illumina P2 adapter was ligated. Finally, the libraries were enriched by high-fidelity PCR amplification (8–12 cycles). RADs for each individual were sequenced on an Illumina HiSeq^TM^ 2500 sequencing platform at Novegene in Beijing, China. Due to the unavailability of existing genomic information for the diploid *L*. *maculatus*, one individual was deep sequenced (approximately 32× coverage) to assemble reliable contigs as a reference assembly for downstream alignment and SNP calling.

### Raw reads filtering and assembly of consensus reference sequences

RAD sequence reads obtained from the Illumina runs were sorted according to individual-specific index sequences. To avoid low-quality reads with artificial bias, raw reads were filtered using the following criteria: 1) removing reads with adapter contamination; 2) reads with ≥ 10% unidentified nucleotides were removed; 3) reads with > 50% bases having phred quality < 5 were removed; 4) putative duplication reads were removed to reduce the impact of PCR artifacts on allele frequency estimation; 5) reads were checked for presence of the partial *Eco*RI motif (^AATTC).

For the reference individual, the remaining first reads with restriction enzyme recognition site after quality control were clustered into RAD cluster tags using cd-hit-est [39]. A maximum of three mismatches between reads was allowed, which corresponded to ~3% of the single-end read length (125bp) [40]. RAD cluster tags with less than 10 or more than 400 reads (approximately 20× of the average read coverage) were discarded. The paired-end reads associated with each RAD cluster tag were extracted and the sequences were sent to the assembly program Velvetopt [41] to construct scaffolds using adjacent contigs identified by paired-end information.

### Read alignment, SNP discovery and filtering

Allowing one permissible alignment per pair read, quality-filtered reads of each individual were aligned to the assembled reference sequences using BWA (version 0.6.2) with default parameters (mismatch penalty 4; gap open penalty 6) [42]. Following the alignment, SNP calling was performed by a conservative Bayesian approach as implemented in the SAMtools package [43]. SNPs were further filtered to maximize data quality according to the following criteria: (i) bi-allelic SNPs; (ii) an average phred score > 20; (iii) coverage depth ≥ 4 and ≤ 100; (iv) missing ratio within each population < 20%; (v) a global minor allele frequency (MAF) ≥ 0.05 in the two pooled populations. Considering the high proportion of paralogous sequence variant (PSVs), only SNPs with *F*_IS_ values between –0.3 and 0.3 and observed heterozygosity values < 0.5 were retained for subsequent analyses [44]; (vi) one SNP was randomly chosen from each RAD tag for subsequent population genetic analyses.

### Outlier tests

Two *F*_ST_-based outlier tests were applied to identify loci that showed divergent patterns of differentiation compared to neutral expectations, and therefore have been potentially affected by selection. First, polymorphic loci were screened for outliers using the coalescent method of Beaumont & Nichols [45] as implemented in LOSITAN [46]. LOSITAN was run using parameter setting of 100 000 simulations, confident interval of 0.995, false discovery rate (FDR) of 0.05, subsample size of 28, attempted *F*_ST_ of 0.055 and simulated *F*_ST_ of 0.052. Second, outlier SNPs were also detected by using the Bayesian simulation approach of Beaumont & Balding [47] as implemented in BAYESCAN [48]. BAYESCAN runs were implemented using default values for all parameters, including a prior odds value of 10, with 100,000 iterations and a burn-in of 50,000 iterations. Loci were considered under selection with a FDR of 0.05.

### Genetic diversity and population differentiation

The VCFtools package [49] was used to estimate observed (*H*_O_) and expected (*H*_E_) heterozygosity for each population. The loci with minimum depth of 4 were generated using ref_map.pl in *Stacks* version 1.32 [50]. Then the nucleotide diversity (π) for each population was calculated by the POPULATIONS program (-r 0.8 -m 4—min_maf 0.05) based on these loci. The whole data set, the neutral SNPs and the putatively adaptive SNPs were used to assess the current distribution of genetic variation by using the Bayesian model-based clustering program of Admixture version 1.2.3 [51]. Furthermore, relationships among individuals within and between populations were calculated and visualized using the NetView P version 0.6 software at a knn = 10 [52]. NetView P is a network analysis pipeline designed for detecting and visualizing complex population structure based on genome-wide SNPs [53]. The VCF files were reformatted with PGDSpider version 2.0.1.1 [54]. *F*_ST_ values between populations based on different datasets were calculated using ARLEQUIN version 3.5.1.3 [55], and significance was determined using 10 000 permutations.

### Population assignment tests

Assignment power of four data sets was evaluated with leave-one-out tests in *GeneClass* version 2.0 [56] to compare the influence of number of SNPs and relative divergence of SNPs on assignment accuracies. These data sets included (i) the complete putative outlier SNPs (298); (ii) 298 randomly chosen SNPs from the complete neutral data set; (iii) 20 randomly chosen SNPs from the complete neutral data set; and (iv) 20 randomly chosen SNPs from the complete putative outlier data set. Individuals were considered to be assigned to a population if the assignment probability to that population was higher than to the other population.

### BLASTX analyses and GO annotation

Contigs containing the outlier SNPs were used as queries in nucleotide searches with BLASTX against the non-redundant protein database of bony fishes at the National Center for Biotechnology Information (NCBI) website (E-value < 1.0E-6). In case of multiple hits, the best match was selected for each outlier containing contig. Gene ontology (GO) functional annotation of the contigs with significant BLASTX hits were obtained using Blast2Go suite (http://www.blast2go.com/b2ghome) [57], which conducts BLAST similarity searches and maps GO terms to the homologous sequences detected. Only ontologies with E-value < 1.0E-6, annotation cut-off > 55 and a GO weight > 5 were considered for annotation.

## Results

### RAD tag sequencing and data filtration

RAD-PE sequencing generated 24.29 million raw read pairs (6.07 G (gigabases) raw data) for the reference individual. After quality filtering, 23.57 million clean read pairs (5.89 G clean data) with the effective rate of 97.03% were retained. After removal of PCR duplicates and only keeping read pairs with the partial *Eco*RI motif (AATTC), 19.50 million reads were finally retained, presenting a clean duplication rate of 11.36% and digestion ratio of 93.35%, respectively (Table 1). For the 29 normally sequenced individuals, sequencing of the RAD libraries generated a total of 169.26 million raw read pairs (45.43 G raw data) (S1 Table). After quality control, a total of 160.8 million clean read pairs (43.18 G clean data) was retained, which presented an average effective rate of 95.0%. Of the retained read pairs, an average of 5.52 million read pairs per individual were kept after removing putative duplication reads and reads without intact *Eco*RI cutting sites (average clean duplication rate of 20.11% and digestion ratio of 95.12%, respectively). Overall, the data showed a high phred quality (phred score 20 ≥ 89.47%; phred score 30 ≥ 81.38%), a stable GC content ranging from 38.67% to 41.7% and a high digestion rate from 76.62% to 98.25%. The Raw RAD-seq reads pairs have been deposited in the Sequence Read Archive database under Accession no. SRP072011.

**Table 1:** Statistics describing the distribution of different properties of each sequenced individual.

Sample	Clean reads	Removed duplication reads	Clean duplication rate (%)	Digestion reads	Digestion ratio (%)
**Reference**					
BHZL7	23,573,826	20,895,973	11.36	19,506,713	93.35
**Beihai**					
BHZL2	6,514,378	5,793,497	11.07	5,482,926	94.64
BHZL3	6,027,708	4,418,570	26.70	3,953,967	89.49
BHZL4	5,528,681	3,421,002	38.12	3,028,070	88.51
BHZL5	4,709,862	3,100,873	34.16	2,656,050	85.65
BHGX10	6,489,759	4,272,832	34.16	4,164,049	97.45
BHGX11	6,482,774	4,588,191	29.22	4,408,480	96.08
BHGX4	5,181,443	4,856,074	6.28	4,498,246	92.63
BHGX8	4,310,893	2,756,430	36.06	2,418,986	87.76
BHGX9	6,484,100	5,895,274	9.08	5,555,381	94.23
BHWS1	7,098,008	6,580,377	7.29	6,167,882	93.73
BHZL1	6,532,443	5,994,190	8.24	5,590,782	93.27
BHZL10	5,874,843	5,493,522	6.49	5,117,905	93.16
BHZL11	6,479,435	5,987,951	7.59	5,625,429	93.95
BHZL8	7,355,495	5,631,322	23.44	5,324,589	94.55
BHZL9	6,482,580	5,930,894	8.51	5,584,640	94.16
**Dandong**					
LNDD1	5,200,570	3,259,943	37.32	2,789,161	85.56
LNDD11	3,436,429	2,303,697	32.96	1,880,963	81.65
LNDD12	6,547,518	6,028,989	7.92	5,727,409	95.00
LNDD13	2,701,201	1,680,911	37.77	1,287,849	76.62
LNDD15	5,970,185	4,599,814	22.95	4,402,605	95.71
LNDD16	6,543,661	5,877,104	10.19	5,704,539	97.06
LNDD17	6,550,388	5,432,012	17.07	5,274,525	97.10
LNDD18	6,525,016	5,226,347	19.90	5,125,077	98.06
LNDD19	6,568,662	5,581,263	15.03	5,483,696	98.25
LNDD20	6,489,129	4,913,880	24.28	4,734,983	96.36
LNDD3	6,551,210	4,222,309	35.55	4,072,621	96.45
LNDD5	6,561,516	5,604,561	14.58	5,427,559	96.84
LNDD6	4,409,786	3,597,430	18.42	3,298,330	91.69
LNDD7	7,101,922	5,113,206	28.00	4,896,347	95.76

### Assembly of the reference sequence

Allowing for a maximum of three mismatches, a total of 3.43 million cluster tags were generated. After removing those cluster tags with less than 10 or more than 400 reads, a total of 223 573 cluster tags containing 15.1 million pair reads were retained. In total, the resulting reference assembly consisted of over 285 408 contigs (~ 113 million nucleotides) with an *N*_50_ size of 509 bp and a GC content of 40.11% (S1 File). After the filtered pair-end reads were realigned onto the assembled contigs, an average depth of 31.56× was obtained and approximately 87.22% of the reference assembly was covered by four or more reads (Table 2).

**Table 2:** Summary statistics of different properties of assembling into reference sequences.

Feature	Value
(i) RAD-PE assembly statistics	
Total contig base (bp)	113,529,353
The number of contigs retained	285,408
Average contig length (bp)	397
N50 contig length (bp)	509
GC content (%)	40.11
(ii) Match statistics	
Mapping rate (%)	90.54
Average depth	31.56
Coverage (> 4×) (%)	87.22
SNP number	217,531

### SNP discovery and analysis

**Fig 1:**
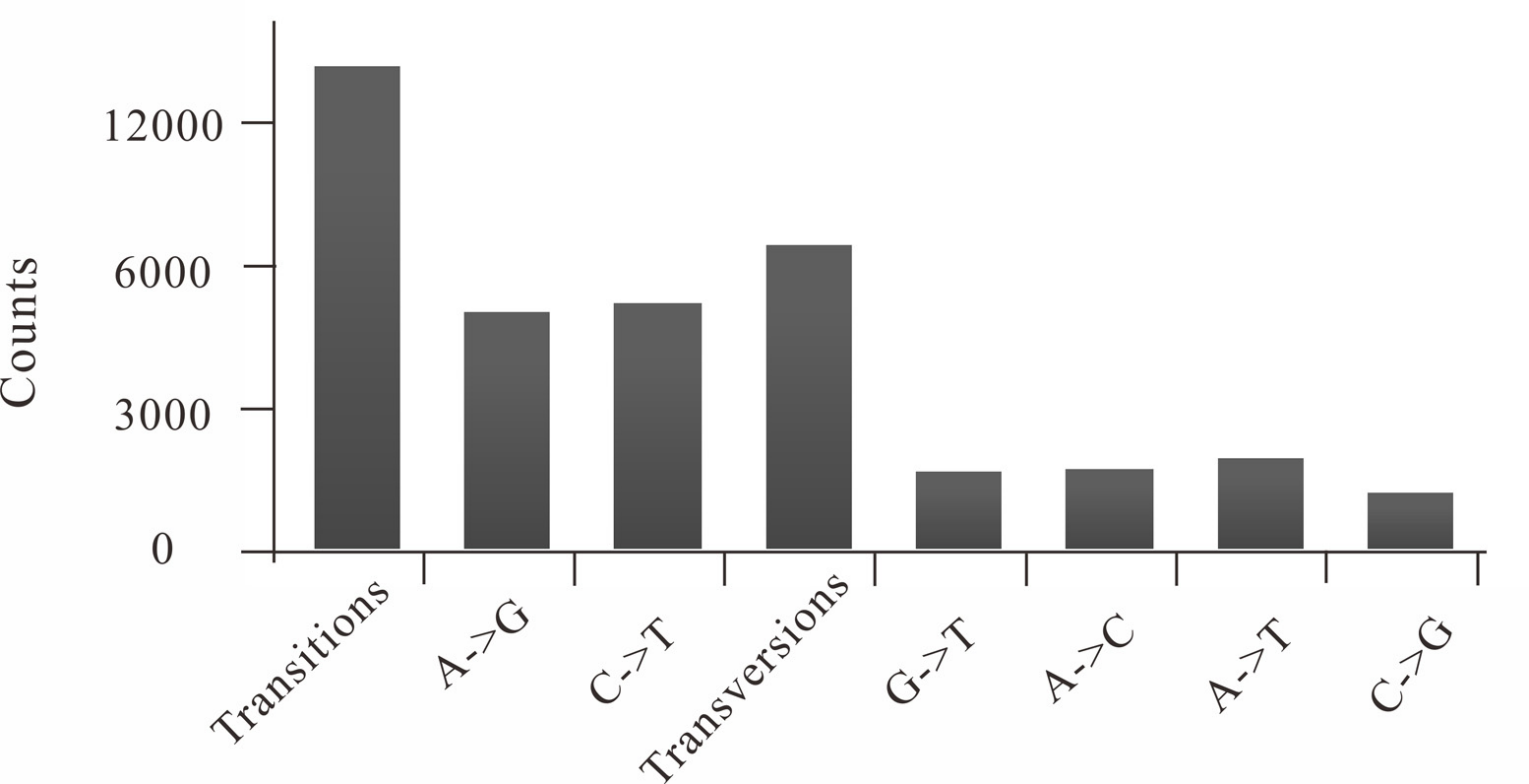
Transitions and transversions occurring within a set of filtered SNPs.

Prior to any quality filtering, a total of 1 184 075 putative SNPs were detected among 30 individuals. After retaining bi-allelic loci with phred score ≥ 20, a total of 1 052 835 SNPs were left. Applying a minimum coverage of four reads and the missing ratio within each population < 20%, a total of 109 307 SNPs were retained. After removing SNPs with a global MAF < 0.05, 64 008 SNPs were left. After only keeping loci with *F*_IS_ values between –0.3 and 0.3 and *H*_O_ < 0.5 in both populations, 42 733 SNPs were finally retained (Table 3; S2 File). The average depth per SNP was above 20 across all sequenced individuals (S2 Table). About 61% of the retained SNPs were proved to be transitions, corresponding to an observed transition / transversion ratio of 1.59 (Fig 1).

**Table 3:** Counts of putative loci after different filtering steps.

Filtering No.	Feature	Value
	Total number of SNPs	1,184,075
i	Bi-allelic SNPs	1,166,783
ii	SNPs with quality score > 20	1,052,835
iii	The average depth of reads > 4 and < 100 and > 80% coverage for each population	109,307
iv	A global minor allele frequency (MAF ≥ 0.05) in two populations	64,008
v	*H*_O_ < 0.5 and -0.3 < *F*_IS_ < 0.3 per SNP for each population	42,733
vi	One SNP per contig	22, 648 (S3 File)

### Outlier detection

**Fig 2:**
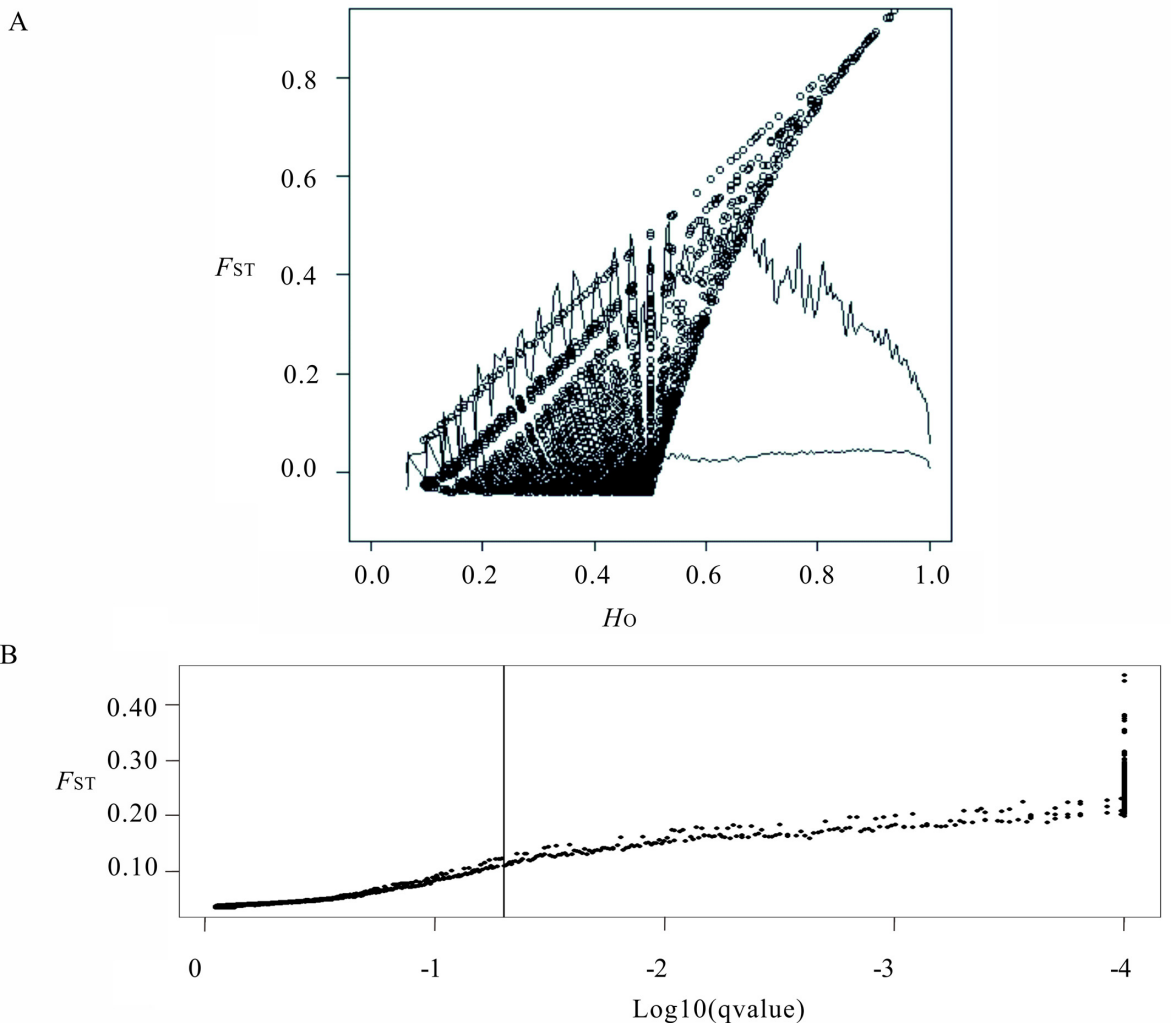
Graphical representation of outlier tests results. (A) results from the LOSITAN. Above the top line is a 0.995 probability for being candidates of selection. A subset of the loci between the two lines is within 0.005–0.995 probability and is considered neutral. The remaining SNPs are conservatively considered undetermined. (B) results from BAYESCAN. The vertical line represents a false discovery threshold of 0.05. The candidate loci under directional selection are on the right side of the vertical line.

A total of 42 733 SNPs were included in both tests for outliers. Using LOSITAN, a total of 3 122 SNPs were identified as outliers possibly under divergent selection after applying a significance level of 0.995. A total of 356 outlier SNPs representing 298 unique contigs were detected by BAYESCAN, all of which were part of those identified using LOSITAN (Fig 2; S4 File).

### Genetic diversity and population structure analysis

For all SNPs, the value of expected heterozygosity (*H*_E_) was 0.3030±0.0945 in Dandong and 0.2807±0.0422 in Beihai. The value of observed heterozygosity (*H*_O_) was 0.3093±0.1246 in Dandong and 0.2781±0.0535 in Beihai. The nucleotide diversity (π) was higher in Dandong (0.0028±0.0001) than in Beihai (0.0018±0.0001). *F*_ST_ values of each SNP varied widely across loci with average of 0.0357, ranging from −0.0535 to 1.

**Fig 3:**
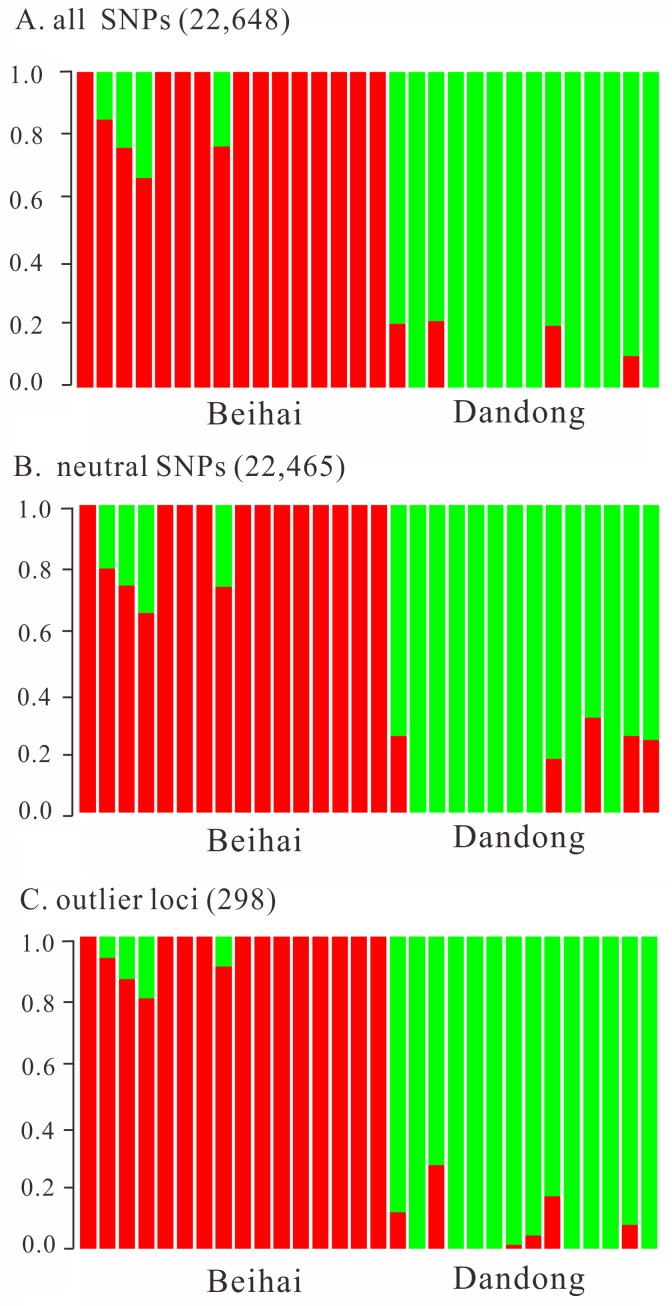
Admixture analysis of *L*. *maculatus* based on all, neutral and outlier SNPs. Each vertical line represents one individual, partitioned into segments according to admixture proportion of the spotted sea bass sampled from Dandong (green) and Beihai (red).

**Fig 4:**
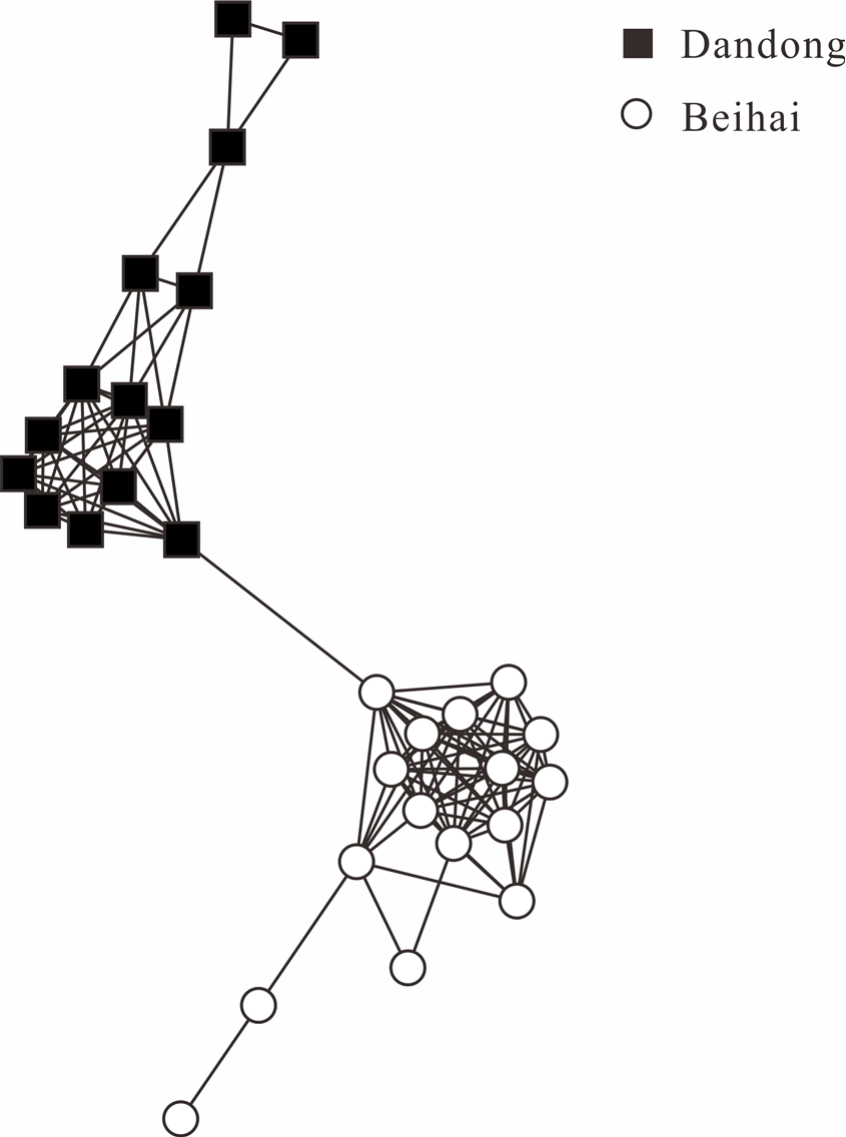
The genetic relationships among individuals of Beihai and Dandong illustrated by the NetView P analysis.

To remove linkage disequilibrium, only one SNP was randomly chosen from each RAD tag for subsequent population genetic analyses, which produced a final data set of 22 648 SNPs. Admixture results based on all three different SNP data sets (whole, neutral, and outlier SNPs) showed that individuals from Dandong and Beihai were clearly separated from each other (Fig 3). Besides, the network of the two populations agreed well with structure detected in the Admixture analyses and genetic break between Beihai and Dandong was clearly visualized in the network topology (Fig 4). *F*_ST_ between the two populations was small but significant based on the whole data set (*F*_ST_ = 0.0550, *P* < 0.001) and neutral SNPs (*F*_ST_ = 0.0347, *P* < 0.001). As expected, *F*_ST_ estimation based on the outlier SNPs yielded a much larger value (*F*_ST_ = 0.6929, *P* < 0.001).

### Population assignment

Assignment accuracy was 100% by using both the complete outlier data set and the equal number of neutral data set. The accuracy based on 20 randomly chosen outlier SNPs,(≥ 93.8%) was higher than that based on 20 randomly chosen neutral SNPs (≥ 78.6%) (Table 4).

**Table 4:** Results of leave-one-out tests for individual assignment with four SNP panels.

Populations	% Correct assignment			
	298 outliers SNPs	298 neutral SNPs	20 outliers SNPs	20 neutral SNPs
Beihai	100	100	93.8	93.8
Dandong	100	100	100	78.6

### BLASTX analysis and GO annotation

**Fig 5:**
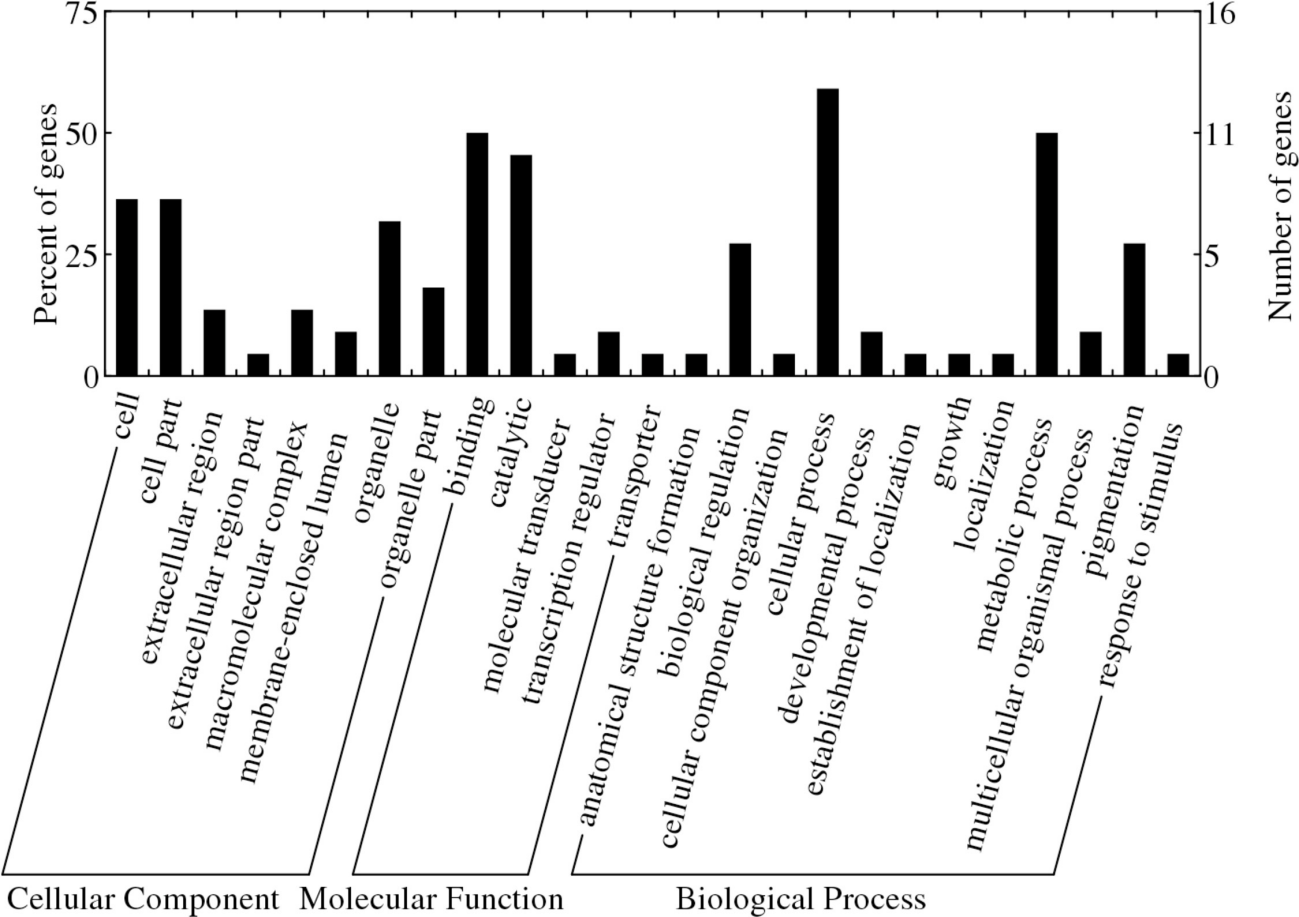
Gene ontology assignment plot. The plot shows GO of candidate genes for adaptive differentiation.

BLASTX analysis of the 298 contigs harboring outlier SNPs against various bony fish genomes resulted in significant hits to 40 fish species. BLASTX similarity results showed that 55 of the 298 contigs corresponded to known proteins in the UniProt database (E-value ≤ 1.0E–6). Functional categorization of the annotated sequences involved in binding and recognition, catalytic and metabolic activities, etc (S3 Table). GO functional annotation of the 55 contigs with significant BLASTX hits yielded GO terms for 22 contigs (40.0%), which were classified into 25 functional groups in three functional categories: molecular function, biological process, and cellular component (Table 5 and Fig 5). Some contigs were classified into more than one functional category, which resulted in the sum of the contig ratio in each category exceeding 100%. Among the contigs categorized as cellular components, 36.67% were classified as cell and 36.67% as cell part. The majority of the contigs categorized as molecular functions was associated with binding (50%) and catalytic activity (41.67%). Most of the contigs categorized as biological process were involved in cellular process (60%) and metabolic process (50%).

**Table 5:** Characterization of 22 GO annotations obtained from Blast2Go analysis.

Contig Name	Description	Length	Hits	min. E-value	mean Similarity	GOs	GOs	Enzyme Codes
16835	mitogen-activated protein kinase 14a-like	476	20	4.79E-10	66.85%	3	F:nucleotide binding; F:protein serine/threonine kinase activity; P:protein phosphorylation	EC:2.7.11
352269	hba1_cotgo ame: full = hemoglobin subunit alpha-1 ame: full = alpha-1-globin ame: full = hemoglobin alpha-1 chain	509	5	2.59E-10	56.80%	4	C:hemoglobin complex; F:oxygen transporter activity; F:metal ion binding; P:oxygen transport	-
2220874	mrg morf4l-binding protein	534	20	2.57E-11	62.15%	2	C:H4/H2A histone acetyltransferase complex; P:regulation of transcription, DNA-templated	-
474018	transcription factor	522	2	4.02E-13	70.50%	4	C:nucleus; C:cytoplasm; F:sequence-specific DNA binding transcription factor activity; P:regulation of transcription from RNA polymerase II promoter	-
3052505	aryl hydrocarbon receptor nuclear translocator-like protein 2 isoform x1	553	20	2.55E-14	90.25%	10	C:nucleus; C:transcription factor complex; C:cytoplasm; F:DNA binding; F:sequence-specific DNA binding transcription factor activity; F:signal transducer activity; F:protein dimerization activity; P:transcription, DNA-templated; P:regulation of transcription, DNA-templated; P:signal transduction	-
2031533	agouti-related	570	20	4.39E-16	71.30%	3	C:extracellular region; F:receptor binding; P:hormone-mediated signaling pathway	-
1205767	r-spondin-2- partial	495	20	9.87E-17	89.90%	3	C:extracellular space; F:G-protein coupled receptor binding; P:positive regulation of canonical Wnt signaling pathway	-
283613	RNA-directed DNA polymerase from transposon BS	586	20	1.93E-17	80.55%	2	F:RNA-directed DNA polymerase activity; P:RNA-dependent DNA replication	EC:2.7.7.49
427869	stam-binding protein	573	20	1.60E-17	77.35%	4	F:metallopeptidase activity; F:pyroglutamyl-peptidase activity; F:metal ion binding; P:proteolysis	EC:3.4.19
2441721	insulin-like growth factor-binding protein 3	497	20	8.30E-20	88.95%	10	C:extracellular region; C:nucleus; F:insulin-like growth factor I binding; F:insulin-like growth factor II binding; P:skeletal system development; P:regulation of cell growth; P:negative regulation of BMP signaling pathway; P:otic vesicle formation; P:insulin-like growth factor receptor signaling pathway; P:pharyngeal system development	-
2174006	terminal uridylyltransferase 4	545	20	4.00E-21	100.00%	4	F:nucleic acid binding; F:zinc ion binding; F:nucleotidyltransferase activity; P:metabolic process	-
3027366_2	PREDICTED: uncharacterized protein LOC103908834	266	20	1.52E-21	63.45%	1	F:transferase activity, transferring glycosyl groups	-
2645499	protein fam50a	368	20	2.00E-23	86.35%	1	C:nucleus	-
1782285	diacylglycerol kinase zeta isoform x1	520	20	3.33E-29	76.95%	3	F:nucleotide binding; F:kinase activity; P:signal transduction	-
136359	Golgi apparatus protein 1	518	20	8.27E-31	98.20%	1	C:Golgi membrane	-
1075810	Golgi apparatus protein 1	570	20	3.47E-32	79.45%	1	C:Golgi membrane	-
2722643	spatacsin	484	20	5.36E-33	79.30%	1	P:axonogenesis	-
1699444	RNA-directed DNA polymerase from mobile element jockey	578	20	9.55E-36	61.60%	2	F:RNA-directed DNA polymerase activity; P:RNA-dependent DNA replication	EC:2.7.7.49
1733564	probable e3 ubiquitin-protein ligase herc1	564	20	1.95E-39	97.25%	3	F:ubiquitin-protein transferase activity; F:ligase activity; P:protein ubiquitination	-
628717	zinc finger protein 423-like isoform x3	580	20	1.58E-41	90.95%	2	F:nucleic acid binding; F:metal ion binding	-
1450955	RNA-directed DNA polymerase from mobile element jockey- partial	626	20	1.08E-79	82.40%	2	F:RNA-directed DNA polymerase activity; P:RNA-dependent DNA replication	EC:2.7.7.49
2312120	reverse transcriptase-like protein	461	20	2.90E-83	68.85%	2	F:RNA-directed DNA polymerase activity; P:RNA-dependent DNA replication	EC:2.7.7.49

## Discussion

In present study, we developed a genome-wide SNP resource of *L*. *maculatus* using RAD-PE method. To our knowledge, this was the first report about the generation of such a large panel of novel SNPs for *L*. *maculatu*s. Furthermore, we highlighted the potential advantages of the genome-wide SNPs for inference of population divergence and candidate adaptive markers detection of *L*. *maculatus*.

### Large-scale SNP identification, genetic diversity, and population genetic structure

As a newly described species from *L*. *japonicus*, the limited number of available molecular markers has constrained population genetic studies of *L*. *maculatus* in the past 10 years. Only 37 polymorphic microsatellites were developed [33, 58]. In addition, the complete mitochondrial genome of *L*. *maculatus* was also available in GenBank [59]. Most previous population genetic studies of *L*. *maculatus* were based on a handful of microsatellite markers, mitochondrial sequence analysis, and random amplified polymorphic DNA (RAPD) markers, which obtained inconsistent results [32, 33, 60, 61].

The transition/transversion ratio was 1.59, which suggested a small influence of sequencing error on calling SNP. Similar transition/transversion ratios have also been observed in the great tit (1.7:1 [62]) and the European eel (1.65:1 [3]). In the absence of a reference genome for *L*. *maculatus*, the contigs generated using paired-end RAD data provided sufficient flanking region around SNPs for design of high-throughput SNP genotyping arrays. This approach has been proved successful for SNP assay design simultaneous with SNP discovery in several studies [38, 63, 64].

The nucleotide diversity was 0.0028 in Dandong and 0.0018 in Beihai. Similar level of variations was identified in the other marine species, such as European eel (π = 0.00529) and small yellow croaker (π = 0.00105) [3, 65]. The higher nuclear genome-wide nucleotide diversity in Dandong than in Beihai was consistent with the results of previous mtDNA study. By using mtDNA control region sequences, Liu *et al*. [32] found that northern populations of *L*. *maculatus* generally showed higher nucleotide diversities than southern ones, with the lowest one found in Beihai. All these results was consistent with the hypotheses that the glacial refugium of *L*. *maculatus* was located in the basin of East China Sea and the genetic diversity is expected to be higher in the ancestral population than in the derived population. Our genome-wide SNP data set demonstrated high power in resolving population genetic structure of *L*. *maculatus*. Both the Structure and NetView P analyses with the whole SNP dataset revealed a clear separation of distinct genetic clusters corresponding to the two geographic populations. However, no genealogical clustering that corresponded to sampling localities was detected by using mtDNA control region sequences [32]. Previous population genetic and phylogeographic studies based on traditional markers demonstrated that most marine fishes generally show low levels or absent of genetic differentiation among geographic regions due to high dispersal potential and an absence of physical barriers [66–68]. The high resolution of genome-wide SNPs has sufficient power to detect population structure even when genetic differentiation is low, as it is typical for marine species. The advantage of genome-wide SNPs over traditional genetic markers in population genetic analyses has been increasingly reported in marine fishes with high gene flow [13–15], which highlighted the utility of genome-wide data in delineating shallow population structure. The genome-wide panel of high quality SNPs generated will facilitate further population genomic and phylogeographic studies on *L*. *maculatus*.

### Population assignment

In the present study, both the putative outlier loci and neutral loci were powerful in population assignment of *L*. *maculatus*. In the past three decades, naturally spawned fry of *L*. *maculatus* were captured from coasts of China, Korea, and Taiwan and distributed to different regions of China, Japan and Korea for cage cultivation [35, 36]. Escaping of cage-cultured *L*. *maculatus* imported from China has been reported in various localities around western Japan, where the spotted sea bass is vigorously cultured [31]. These new informative SNPs, especially the outliers, would be useful for increasing accuracy when assigning individual *L*. *maculatus* to population-of-origin in aquaculture using naturally spawned fry, which would facilitate the scientific management and sustainable exploitation of the genetic resource of natural populations of *L*. *maculatus*. Since the two populations analyzed in the present study were geographically distant and genetically differentiated, screening of further samples from geographically close localities will be required to assess the accuracy reported in this study. Non-neutral markers can be useful for individual and composition assignment [69]. Indeed, the 20 randomly chosen outlier SNPs performed better than the 20 neutral SNPs in *L*. *maculatus*. Outlier loci have also been proved successful for individual and compositional assignment in various fishes. For example, Larson *et al*. [18] demonstrated that outliers identified by RAD-seq in Chinook salmon (*Oncorhynchus tshawytscha*) can be used to create high-resolution panels for genetic monitoring and population assignment.

### Local adaptation

Recently, the advent of high-throughput DNA sequencing technology provides a novel approach for investigating local adaptation in natural populations of marine fishes [14, 18,70]. BLASTX analyses of the outlier-containing contig sequences revealed that only 55 out of 298 (18.4%) highly divergent contigs were located in functional genes or genomic regions, suggesting that most of the putative outlier SNPs detected in *L*. *maculatus* were located in unknown proteins and non-coding genomic regions influenced by selection through genetic hitchhiking [48]. The BLASTX annotated contigs in the present study are involved in metabolism, growth, immunity and biorhythm. Contig_1733564 was annotated as an E3 ubiquitin-protein ligase gene (*HERC1*), which is involved in the ubiquitin mediated proteolysis. contig_1782285 (diacyllycerol kinase zeta isoform x1 gene, *DGKZ*) is a gene involved in pathways for glycerolipid metabolism and glycerophospholipid metabolism. Contig_612117 (C-terminal binding protein gene, *CtBP*) is a key transcriptional coregulator in adipose tissue, which works with several different partner proteins to regulate the development of both white and brown adipocytes [71]. Contig_1242038 (lipase maturation factor 2 gene, *LMF2*) may be required for maturation and transport of active lipoprotein lipase through the secretary pathway. Contig_2602294 (death-associated protein kinase 1-like gene, *DAPK1*) is an important regulator of the cellular antiviral response [72]. Contig_3052505 was annotated as aryl hydrocarbon receptor nuclear translocator-like 2 (*ARNTL*2), which is an essential component within the clock gene regulatory network. Contig_628717, contig_2583004, contig_432419, and contig_525464 were annotated as zinc finger protein gene (*ZNF*), which was reported to play broad-spectrum cellular functions in eukaryotic cells biology [73]. Meanwhile, other studies of marine fishes also found the same or similar functional candidate genes potentially important for local adaptation, such as transcription factor (contig_474018), Golgi apparatus protein (contig_1075810) [70] and zinc finger protein, RNA-directed DNA polymerase from mobile element jockey (contig_1450955; contig_1699444), RNA-directed DNA polymerase from mobile element jockey-like (contig_105161), RNA-directed DNA polymerase from transposon BS (contig_283613) [65]. The consistent results suggested that these candidate genes may play important roles in local adaptation. Moreover, GO functional annotation of 22 out of the 55 contigs with significant BLASTX hits demonstrated that the majority of the contigs categorized as molecular functions was associated with binding and catalytic activity, and most of the contigs categorized as biological process were involved in cellular process and metabolic process, indicating that these outliers are likely to be biologically relevant for adaptation of populations to local environments. Species that occupy heterogeneous environments (i.e. temperature) along their geographical distribution experience spatially varying selective pressure, which often result in local adaptation of ecologically important traits [74]. The two *L*. *maculatus* populations were collected from the Yellow Sea and the South China Sea with highly heterogeneous environments. Indeed, variance in ecologically important life history traits such as growth rate, size at maturity and spawning season have been observed among populations of *L*. *maculatus* [75, 76]. Since *L*. *maculatus* re-colonized the extensive continental shelf of the China sea from glacial refugium in the East China Sea after the Last Glacial Maximum (LGM [32]), these putative adaptive outliers suggested that natural populations adapt to local environments could have occurred after LGM. Guo *et al*. [70] analyzed > 30 000 SNPs based on a pooled RAD-seq approach from 10 populations of Baltic three-spined sticklebacks and provided strong evidence for heterogenic genomic divergence driven by local adaptation along an environmental gradient in this postglacial ecosystem. We recommend that further population genomic studies use multi-populations across the distribution of *L*. *maculatus* and couple the allele frequencies with environmental data to pinpoint regions of the *L*. *maculatus* genome under selection.

## Supporting Information

S1 Table Summary of the sequencing parameters for each individual. (DOCX)

S2 Table Summary statistics of SNPs detected in each individual. (DOCX)

S3 Table A list of the 55 best-quality BLASTx matches with E-value < 1E-6. (DOCX)

S1 File The sequence assembly file. (7Z)

S2 File The whole filtered SNP dataset. (VCF)

S3 File The filtered SNP data file, one SNP for each contig. (VCF)

S4 File The outlier SNP dataset t. (VCF)

## References

[1] Thurstan RH, Brockington S, Roberts CM. The effects of 118 years of industrial fishing on UK bottom trawl fisheries. Nature communications, 2010; 1:15 10.1038/ncomms1013 20975682

[2] Reiss H, Hoarau G, Dickey-Collas M, Wolff WJ. Genetic population structure of marine fish: mismatch between biological and fisheries management units. Fish and Fisheries, 2009; 10(4): 361–395. 10.1111/j.1467-2979.2008.00324.x

[3] Pujolar JM, Jacobsen MW, Frydenberg J, Als TD, Larsen PF, Maes GE, et al A resource of genome-wide single-nucleotide polymorphisms generated by RAD tag sequencing in the critically endangered European eel. Molecular Ecology Resources, 2013; 13(4): 706–714. 10.1111/1755-0998.12117 23656721

[4] Utter F, Ryman N. Genetic markers and mixed stock fisheries. Fisheries, 1993; 18(8): 11–21. 10.1577/1548-8446(1993)018<0011:GMAMSF>2.0.CO;2

[5] Shaklee JB, Beacham TD, Seeb L, White BA. Managing fisheries using genetic data: case studies from four species of Pacific salmon. Fisheries Research, 1999; 43(1): 45–78.

[6] Waples RS, Dickhoff WW, Hauser L, Ryman N. Six decades of fishery genetics: taking stock. Fisheries, 2008; 33: 76–79.

[7] Allendorf FW, Hohenlohe PA, Luikart G. Genomics and the future of conservation genetics. Nature Reviews Genetics, 2010; 11(10): 697–709. 10.1038/nrg2844 20847747

[8] Avise JC. Perspective: conservation genetics enters the genomics era. Conservation Genetics, 2010; 11(2): 665–669. 10.1007/s10592-009-0006-y

[9] Narum SR, Buerkle CA, Davey JW, Miller MR, Hohenlohe PA. Genotyping-by-sequencing in ecological and conservation genomics. Molecular Ecology, 2013; 22(11): 2841–2847. 10.1111/mec.12350 23711105 PMC3935057

[10] Shimada Y, Shikano T, Merila J. A high incidence of selection on physiologically important genes in the three-spined stickleback, *Gasterosteus aculeatus*. Molecular Biology and Evolution, 2011; 28(1): 181–193. 10.1093/molbev/msq181 20660084

[11] Angeloni F, Wagemaker N, Vergeer P, Ouborg J. Genomic toolboxes for conservation biologists. Evolutionary Applications, 2012; 5(2): 130–143. 10.1111/j.1752-4571.2011.00217.x 25568036 PMC3353346

[12] Savolainen O, Lascoux M, Merila J. Ecological genomics of local adaptation. Nature Reviews Genetics, 2013; 14(11): 807–820. 10.1038/nrg3522 24136507

[13] Hess JE, Campbell NR, Close DA, Docker MF, Narum SR. Population genomics of Pacific lamprey: adaptive variation in a highly dispersive species. Molecular Ecology, 2013; 22(11): 2898–2916. 10.1111/mec.12150 23205767

[14] Bourret V, Kent MP, Primmer CR, Vasemagi A, Karlsson S, Hindar K, et al SNP-array reveals genome-wide patterns of geographical and potential adaptive divergence across the natural range of Atlantic salmon (*Salmo salar*). Molecular Ecology, 2013; 22(3): 532–551. 10.1111/mec.12003 22967111

[15] Pujolar JM, Jacobsen MW, Als TD, Frydenberg J, Munch K, Jonsson B, et al Genome-wide single-generation signatures of local selection in the panmictic European eel. Molecular Ecology, 2014; 23(10): 2514–2528. 10.1111/mec.12753 24750353

[16] Hohenlohe PA, Bassham S, Etter PD, Stiffler N, Johnson EA, Cresko WA. Population genomics of parallel adaptation in threespine stickleback using sequenced RAD tags. PLoS Genetics, 2010; 6(2): e1000862 10.1371/journal.pgen.1000862 20195501 PMC2829049

[17] Russello MA, Kirk SL, Frazer KK, Askey PJ. Detection of outlier loci and their utility for fisheries management. Evolutionary Applications, 2012; 5(1): 39–52. 10.1111/j.1752-4571.2011.00206.x 25568028 PMC3353332

[18] Larson WA, Seeb LW, Everett MV, Waples RK, Templin WD, Seeb JE. Genotyping by sequencing resolves shallow population structure to inform conservation of Chinook salmon (*Oncorhynchus tshawytscha*). Evolutionary Applications, 2014; 7(3): 355–369. 10.1111/eva.12128 24665338 PMC3962296

[19] Hemmer-Hansen J, Nielsen EE, Therkildsen NO, Taylor MI, Ogden R, Geffen AJ, et al A genomic island linked to ecotype divergence in Atlantic cod. Molecular Ecology, 2013; 22(10): 2653–2667. 10.1111/mec.12284 23611647

[20] Vandamme SG, Maes GE, Raeymaekers JA, Cottenie K, Imsland AK, Hellemans B, et al Regional environmental pressure influences population differentiation in turbot (*Scophthalmus maximus*). Molecular Ecology, 2014; 23(3): 618–636. 10.1111/mec.12628 24354713

[21] Baird NA, Etter PD, Atwood TS, Currey MC, Shiver AL, Lewis ZA, et al Rapid SNP discovery and genetic mapping using sequenced RAD markers. PloS One, 2008; 3(10): e3376 10.1371/journal.pone.0003376 18852878 PMC2557064

[22] Van Tassell CP, Smith TP, Matukumalli LK, Taylor JF, Schnabel RD, Lawley CT, et al SNP discovery and allele frequency estimation by deep sequencing of reduced representation libraries. Nature Methods, 2008; 5(3): 247–252. 10.1038/nmeth 18297082

[23] Maughan PJ, Yourstone SM, Jellen EN, Udall JA. SNP discovery via genomic reduction, barcoding, and 454-pyrosequencing in amaranth. The Plant Genome, 2009; 2(3): 260–270. 10.3835/plantgenome2009.08.0022

[24] Fu YB, Peterson GW. Developing genomic resources in two Linum species via 454 pyrosequencing and genomic reduction. Molecular Ecology Resources, 2012; 12(3): 492–500. 10.1111/j.1755-0998.2011.03100.x 22177006

[25] Sanchez CC, Smith TP, Wiedmann RT, Vallejo RL, Salem M, Yao J, et al Single nucleotide polymorphism discovery in rainbow trout by deep sequencing of a reduced representation library. BMC Genomics, 2009; 10:559 10.1186/1471-2164-10-559 19939274 PMC2790473

[26] Gompert Z, Forister ML, Fordyce JA, Nice CC, Williamson RJ, Buerkle CA. Bayesian analysis of molecular variance in pyrosequences quantifies population genetic structure across the genome of *Lycaeides* butterflies. Molecular Ecology, 2010; 19(12): 2455–2473. 10.1111/j.1365-294X.2010.04666.x 20497324

[27] Seeb JE, Carvalho G, Hauser L, Naish K, Roberts S, Seeb LW. Single-nucleotide polymorphism (SNP) discovery and applications of SNP genotyping in nonmodel organisms. Molecular Ecology Resources, 2011; 11(Suppl 1): 1–8. 10.1111/j.1755-0998.2010.02979.x 21429158

[28] Nelson JS Fishes of the world, 4th ed. Hobken, NJ: John Wiley & Sons, Inc. 2006; ISBN 978-0-471-25031-9.

[29] Niu S, Liu Y, Qin C, Wang X, Wu R. The complete mitochondrial genome and phylogenetic analysis of *Lateolabrax maculatus* (Perciformes, Moronidae). Mitochondrial DNA, 2015; 10.3109/19401736.2015.1115496 26709754

[30] Kim Y, Myoung J, Kim Y, Han K, Kang C, Kim J, et al The marine fishes of Korea. Hanguel, Pusan. 2001: 222.

[31] Yokogawa K, Taniguchi N, Seki S. Morphological and Genetic Differences between Japanese and Chinese Sea Bass of the Genus *Lateolabrax*. Japanese Journal of Ichthyology, 1995; 41(4): 437–445.

[32] Liu JX, Gao TX, Yokogawa K, Zhang YP. Differential population structuring and demographic history of two closely related fish species, Japanese sea bass (*Lateolabrax japonicus*) and spotted sea bass (*Lateolabrax maculatus*) in Northwestern Pacific. Molecular Phylogenetics and Evolution, 2006; 39(3): 799–811. 10.1016/j.ympev.2006.01.009 16503171

[33] Jiang X, Yang G, Liao M, Liu Y, Gao T, Wang D, et al Microsatellite DNA polymorphism of Japanese sea bass (*Laterolabrax japonicus*) inhabiting Chinese and Japanese coasts. Journal of Applied Ichthyology, 2008; 24(2): 180–186. 10.1111/j.1439-0426.2007.01016.x

[34] An HS, Lee JW, Kim HY, Kim JB, Chang DS, Park JY, et al Genetic differences between wild and hatchery populations of Korean spotted sea bass (*Lateolabrax maculatus*) inferred from microsatellite markers. Genes & Genomics, 2013; 35(5): 671–680. 10.1007/s13258-013-0135-z

[35] Matsuoka S. History and present situation of marine fin-fish culture at Ehime Prefecture. Suisanzoshoku, 1993; 41: 265–271. (In Japanese with English abstract).

[36] Chen DG, Gao TX, Zeng XQ, Ren YP, Ruan SH. Study on the fishery biology of Laizhou population of *Lateolabrax* sp. Acta Oceanologica Sinica, 2001; 23: 81–86. (In Chinese with English abstract)

[37] Sambrook J, Russell DW. Molecular cloning: A laboratory manual, 3^rd^ ed. 2001, Cold Spring Harbor Laboratory Press, New York.

[38] Etter PD, Preston JL, Bassham S, Cresko WA, Johnson EA. Local *de novo* assembly of RAD paired-end contigs using short sequencing reads. PloS One, 2011; 6(4): e18561 10.1371/journal.pone.0018561 21541009 PMC3076424

[39] Li W, Godzik A. Cd-hit: a fast program for clustering and comparing large sets of protein or nucleotide sequences. Bioinformatics, 2006; 22(13): 1658–1659. 10.1093/bioinformatics/btl158 16731699

[40] Ilut DC, Nydam ML, Hare MP. Defining loci in restriction-based reduced representation genomic data from nonmodel species: sources of bias and diagnostics for optimal clustering. BioMed Research International, 2014; vol.2014: Article ID 675158. 10.1155/2014/675158 PMC4095725 25057498

[41] Zerbino DR, Birney E. Velvet: algorithms for *de novo* short read assembly using de Bruijn graphs. Genome Research, 2008; 18(5): 821–829. 10.1101/gr.074492.107 18349386 PMC2336801

[42] Li H, Durbin R. Fast and accurate short read alignment with Burrows-Wheeler transform. Bioinformatics, 2009; 25(14): 1754–1760. 10.1093/bioinformatics/btp324 19451168 PMC2705234

[43] Li H, Handsaker B, Wysoker A, Fennell T, Ruan J, Homer N, et al The Sequence Alignment/Map format and SAMtools. Bioinformatics, 2009; 25(16): 2078–2079. 10.1093/bioinformatics/btp352 19505943 PMC2723002

[44] Hohenlohe PA, Amish SJ, Catchen JM, Allendorf FW, Luikart G. Next-generation RAD sequencing identifies thousands of SNPs for assessing hybridization between rainbow and westslope cutthroat trout. Molecular Ecology Resources, 2011; 11 (Suppl. 1): 117–122. 10.1111/j.1755-0998.2010.02967.x 21429168

[45] Beaumont MA, Nichols RA. Evaluating loci for use in the genetic analysis of population structure. Proceedings of the Royal Society of London B: Biological Sciences, 1996; 263(1377): 1619–1626. 10.1098/rspb.1996.0237

[46] Antao T, Lopes A, Lopes RJ, Beja-Pereira A, Luikart G. LOSITAN: a workbench to detect molecular adaptation based on a *F*_ST_-outlier method. BMC Boinformatics, 2008; 9: 323 10.1186/1471-2105-9-323 PMC2515854 18662398

[47] Beaumont MA, Balding DJ. Identifying adaptive genetic divergence among populations from genome scans. Molecular Ecology, 2004; 13(4): 969–980. 10.1111/j.1365-294X.2004.02125.x 15012769

[48] Foll M, Gaggiotti O. A genome-scan method to identify selected loci appropriate for both dominant and codominant markers: a Bayesian perspective. Genetics, 2008; 180(2): 977–993. 10.1534/genetics.108.092221 18780740 PMC2567396

[49] Danecek P, Auton A, Abecasis G, Albers CA, Banks E, DePristo MA, et al The variant call format and VCFtools. Bioinformatics, 2011; 27(15): 2156–2158. 10.1093/bioinformatics/btr330 21653522 PMC3137218

[50] Catchen J, Hohenlohe PA, Bassham S, Amores A, Cresko WA. Stacks: an analysis tool set for population genomics. Molecular Ecology, 2013; 22(11): 3124–3140. 10.1111/mec.12354 23701397 PMC3936987

[51] Alexander DH, Novembre J, Lange K. Fast model-based estimation of ancestry in unrelated individuals. Genome Research, 2009; 19(9): 1655–64. 10.1101/gr.094052 19648217 PMC2752134

[52] Steinig EJ, Neuditschko M, Khatkar MS, Raadsma HW, Zenger KR. NetView P: a network visualization tool to unravel complex population structure using genome-wide SNPs. Molecular Ecology Resources, 2016; 16: 216–227. 10.1111/1755-0998.12442 26129944

[53] Neuditschko M, Khatkar MS, Raadsma HW. NetView: a high-definition network-visualization approach to detect fine-scale population structures from genome-wide patterns of variation. PloS One, 2012; 7(10): e48375 10.1371/journal.pone.0048375 23152744 PMC3485224

[54] Lischer HE, Excoffier L. PGDSpider: an automated data conversion tool for connecting population genetics and genomics programs. Bioinformatics, 2012; 28(2): 298–299. 10.1093/bioinformatics/btr642 22110245

[55] Excoffier L, Lischer HE. Arlequin suite ver 3.5: a new series of programs to perform population genetics analyses under Linux and Windows. Molecular Ecology Resources, 2010 10(3): 564–567. 10.1111/j.1755-0998.2010.02847.x 21565059

[56] Piry S, Alapetite A, Cornuet JM, Paetkau D, Baudouin L, Estoup A. GENECLASS2: a software for genetic assignment and first-generation migrant detection. Journal of Heredity, 2004; 95(6): 536–539. 10.1093/jhered/esh074 15475402

[57] Gotz S, Garcia-Gomez JM, Terol J, Williams TD, Nagaraj SH, Nueda MJ, et al High-throughput functional annotation and data mining with the Blast2Go suite. Nucleic Acids Research, 2008; 36(10): 3420–3435. 10.1093/nar/gkn176 18445632 PMC2425479

[58] Zhao Y, Ji XS, Zeng YQ, Ding L, Yang PP, Wang H. Isolation of microsatellite markers for *Lateolabrax japonicus* and polymorphic analysis. Zoological Research, 2011; 32(5): 515–520. 10.3724/SP.J.1141.2011.05515 (In Chinese with English abstract) 22006804

[59] Bi YH, Chen XW. Mitochondrial genome of the Japanese seabass *Lateolabrax japonicus* (Teleostei, Perciformes, and Moronidae). Mitochondrial DNA, 2012;23(5):371–372. 10.3109/19401736.2012.696630 22803710

[60] Hu ZM, Gao TX, Han ZQ, Song L. Studies on Genetic Differentiation of the Spotted Sea Bass (*Lateolabrax maculatus*) and Japanese Sea Bass (*Lateolabrax japonicus*). Periodical of Ocean University of China, 2007; 3: 413–418. (In Chinese with English abstract)

[61] Liu MY, Jiang QC, Yang JX. Analysis on Mitochondrial DNA Cytochrome b gene of *Lateolabrax japonicus* from different seas. Journal of Nanjing Normal University, 2010; 33(1): 102–106. (In Chinese with English abstract)

[62] van Bers NE, van Oers K, Kerstens HH, Dibbits BW, Crooijmans RP, Visser ME, et al Genome-wide SNP detection in the great tit *Parus major* using high throughput sequencing. Molecular Ecology, 2010; 19(Suppl. 1): 89–99. 10.1111/j.1365-294X.2009.04486.x 20331773

[63] Willing EM, Hoffmann M, Klein JD, Weigel D, Dreyer C. Paired-end RAD-seq for *de novo* assembly and marker design without available reference. Bioinformatics, 2011; 27(16): 2187–2193. 10.1093/bioinformatics/btr346 21712251

[64] Hohenlohe PA, Day MD, Amish SJ, Miller MR, Kamps-Hughes N, Boyer MC, et al Genomic patterns of introgression in rainbow and westslope cutthroat trout illuminated by overlapping paired-end RAD sequencing. Molecular Ecology, 2013; 22(11): 3002–3013. 10.1111/mec.12239 23432212 PMC3664261

[65] Zhang BD, Xue DX, Wang J, Li YL, Liu BJ, Liu JX. Development and preliminary evaluation of a genomewide single nucleotide polymorphisms resource generated by RAD-seq for the small yellow croaker (*Larimichthys polyactis*). Molecular Ecology Resources, 2016; 16: 755–768. 10.1111/1755-0998.12476 26439680

[66] Palumbi SR. Genetic divergence, reproductive isolation, and marine speciation. Annual Review of Ecology and Systematics, 1994; 25: 547–572. 10.1146/annurev.es.25.110194.002555

[67] Grant W, Bowen B. Shallow population histories in deep evolutionary lineages of marine fishes: insights from sardines and anchovies and lessons for conservation. Journal of Heredity, 1998; 89(5): 415–426. 10.1093/jhered/89.5.415

[68] Hewitt G. The genetic legacy of the Quaternary ice ages. Nature, 2000; 405: 907–913. 10.1038/35016000 10879524

[69] Freamo H, O’REILLY P, Berg PR, LIEN S, Boulding EG. Outlier SNPs show more genetic structure between two Bay of Fundy metapopulations of Atlantic salmon than do neutral SNPs. Molecular Ecology Resources, 2011; 11 (Suppl. 1): 254–267. 10.1111/j.1755-0998.2010.02952.x 21429179

[70] Guo B, DeFaveri J, Sotelo G, Nair A, Merila J. Population genomic evidence for adaptive differentiation in Baltic Sea three-spined sticklebacks. BMC Biology, 2015;13:19 10.1186/s12915-015-0130-8 25857931 PMC4410466

[71] Jack BHA, Pearson RC, Crossley M. C-terminal binding protein: A metabolic sensor implicated in regulating adipogenesis. The International Journal of Biochemistry & Cell Biology, 2011; 43: 693–696. 10.1016/j.biocel.2011.01.017 21281737

[72] Zhang J, Hu MM, Shu HB, Li S. Death-associated protein kinase 1 is an IRF3/7-interacting protein that is involved in the cellular antiviral immune response. Cellular & Molecular Immunology, 2014; 11: 245–252. 10.1038/cmi.2013.65 24531619 PMC4085485

[73] Laity JH, Lee BM, Wright PE. Zinc finger proteins: new insights into structural and functional diversity. Current Opinion in Structural Biology, 2001; 11: 39–46. 10.1016/S0959-440X(00)00167-6 11179890

[74] Fraser HB, Babak T, Tsang J, Zhou Y, Zhang B, Mehrabian M, et al Systematic detection of polygenic cis-regulatory evolution. PLoS Genetics, 2011; 7(3): e1002023 10.1371/journal.pgen.1002023 21483757 PMC3069120

[75] Liao G. The ecological characteristics and pond farming problems of *Lateolabrax maculatus*. Fisheries Science & Technology Information, 1998; 25(3): 130–132. (In Chinese with English abstract)

[76] Wang Y, Lv Z, Gao T, Zheng G, Zhang W. Comparative analysis of nutritional components of *Lateolabrax* sp. in different sea areas. Journal of Ocean University of Qingdao, 2003; 4: 531–536. (In Chinese with English abstract)

