# Supplementary material for: Genome-Wide SNP Discovery, Genotyping and Their Preliminary Applications for Population Genetic Inference in Spotted Sea Bass (Lateolabrax maculatus)
Source: PLoS One. 2016 Jun 23;11(6):e0157809. doi: 10.1371/journal.pone.0157809 (PMC4919078; doi:10.1371/journal.pone.0157809)
Supplement: S1 Table — (DOCX) [file pone.0157809.s001.docx]

**S1** **Table. Summary of the sequencing parameters for each individual.**

| **Sample** | **Raw Base (bp)** | **Clean Base (bp)** | **Effective Rate (%)** | **Error Rate (%)** | **Q20 (%)** | **Q30 (%)** | **GC Content (%)** |
| --- | --- | --- | --- | --- | --- | --- | --- |
| **Reference** |  |  |  |  |  |  |  |
| BHZL7 | 6,074,148,250 | 5,893,456,500 | 97.03 | 0.05 | 90.73 | 83.38 | 39.27 |
| **Pop BH** |  |  |  |  |  |  |  |
| BHZL2 | 1,687,500,000 | 1,628,594,500 | 96.51 | 0.06 | 90.73 | 83.65 | 41.00 |
| BHZL3 | 1,634,818,750 | 1,506,927,000 | 92.18 | 0.04 | 92.38 | 86.38 | 40.71 |
| BHZL4 | 1,500,000,000 | 1,382,170,250 | 92.14 | 0.04 | 94.50 | 90.03 | 39.08 |
| BHZL5 | 1,313,789,500 | 1,177,465,500 | 89.62 | 0.04 | 93.00 | 87.50 | 39.51 |
| BHGX10 | 1,687,500,000 | 1,622,439,750 | 96.14 | 0.04 | 93.97 | 88.86 | 40.95 |
| BHGX11 | 1,687,500,000 | 1,620,693,500 | 96.04 | 0.04 | 92.17 | 85.93 | 40.60 |
| BHGX4 | 1,360,207,250 | 1,295,360,750 | 95.23 | 0.06 | 90.30 | 82.69 | 40.27 |
| BHGX8 | 1,179,106,000 | 1,077,723,250 | 91.40 | 0.04 | 93.63 | 88.52 | 40.71 |
| BHGX9 | 1,687,500,000 | 1,621,025,000 | 96.06 | 0.05 | 91.29 | 84.42 | 39.03 |
| BHWS1 | 1,840,571,750 | 1,774,502,000 | 96.41 | 0.06 | 90.56 | 83.06 | 39.49 |
| BHZL1 | 1,702,148,500 | 1,633,110,750 | 95.94 | 0.05 | 90.69 | 83.41 | 39.08 |
| BHZL10 | 1,531,845,750 | 1,468,710,750 | 95.88 | 0.06 | 90.00 | 82.17 | 40.41 |
| BHZL11 | 1,687,500,000 | 1,619,858,750 | 95.99 | 0.04 | 91.55 | 84.88 | 38.67 |
| BHZL8 | 1,918,580,000 | 1,838,873,750 | 95.85 | 0.04 | 94.70 | 90.25 | 40.90 |
| BHZL9 | 1,687,500,000 | 1,620,645,000 | 96.04 | 0.05 | 91.32 | 84.42 | 38.86 |
| **Pop DD** |  |  |  |  |  |  |  |
| LNDD1 | 1,444,357,000 | 1,300,142,500 | 90.02 | 0.04 | 92.28 | 86.23 | 41.00 |
| LNDD11 | 995,490,250 | 859,107,250 | 86.30 | 0.05 | 92.05 | 85.84 | 40.60 |
| LNDD12 | 1,687,500,000 | 1,636,879,500 | 97.00 | 0.05 | 90.82 | 83.91 | 40.35 |
| LNDD13 | 839,693,250 | 675,300,250 | 80.42 | 0.04 | 92.50 | 86.69 | 39.94 |
| LNDD15 | 1,543,054,000 | 1,492,546,250 | 96.73 | 0.04 | 92.25 | 85.93 | 40.09 |
| LNDD16 | 1,687,500,000 | 1,635,915,250 | 96.94 | 0.04 | 92.63 | 86.50 | 40.04 |
| LNDD17 | 1,687,500,000 | 1,637,597,000 | 97.04 | 0.04 | 94.28 | 89.38 | 40.31 |
| LNDD18 | 1,687,500,000 | 1,631,254,000 | 96.67 | 0.04 | 93.90 | 88.72 | 40.85 |
| LNDD19 | 1,687,500,000 | 1,642,165,500 | 97.31 | 0.04 | 93.77 | 88.56 | 40.36 |
| LNDD20 | 1,687,500,000 | 1,622,282,250 | 96.14 | 0.04 | 93.97 | 88.89 | 40.92 |
| LNDD3 | 1,687,500,000 | 1,637,802,500 | 97.05 | 0.04 | 92.83 | 86.77 | 41.00 |
| LNDD5 | 1,687,500,000 | 1,640,379,000 | 97.21 | 0.04 | 92.65 | 86.61 | 40.12 |
| L NDD6 | 1,167,645,750 | 1,102,446,500 | 94.42 | 0.07 | 89.47 | 81.38 | 41. 70 |
| LNDD7 | 1,831,566,500 | 1,775,480,500 | 96.94 | 0.05 | 92.12 | 85.69 | 40.49 |
