# Supplementary material for: Genome-Wide SNP Discovery, Genotyping and Their Preliminary Applications for Population Genetic Inference in Spotted Sea Bass (Lateolabrax maculatus)
Source: PLoS One. 2016 Jun 23;11(6):e0157809. doi: 10.1371/journal.pone.0157809 (PMC4919078; doi:10.1371/journal.pone.0157809)
Supplement: S2 Table — (DOCX) [file pone.0157809.s002.docx]

**S2 Table. Summary statistics of SNPs detected in each individual.**

| **Sample** | **Total Number** | **Hom.** | **Het.** | **Total Depth** | **Average Depth** |
| --- | --- | --- | --- | --- | --- |
| **BH** |  |  |  |  |  |
| BHZL2 | 27,550 | 14,211 | 13,339 | 707,341 | 25.67 |
| BHZL3 | 28,449 | 13,831 | 14,618 | 582,972 | 20.49 |
| BHZL4 | 28,735 | 13,136 | 15,599 | 383,360 | 13.34 |
| BHZL5 | 29,648 | 12,624 | 17,024 | 356,214 | 12.01 |
| BHGX10 | 26,811 | 14,042 | 12,769 | 618,696 | 23.08 |
| BHGX11 | 27,086 | 14,412 | 12,674 | 634,747 | 23.43 |
| BHGX4 | 28,056 | 13,891 | 14,165 | 557,028 | 19.85 |
| BHGX8 | 27,993 | 12,235 | 15,758 | 371,355 | 13.27 |
| BHGX9 | 27,808 | 14,102 | 13,706 | 657,461 | 23.64 |
| BHWS1 | 27,886 | 14,140 | 13,746 | 727,969 | 26.11 |
| BHZL10 | 28,026 | 14,006 | 14,020 | 666,766 | 23.79 |
| BHZL11 | 27,856 | 14,053 | 13,803 | 670,052 | 24.05 |
| BHZL1 | 27,949 | 14,017 | 13,932 | 660,699 | 23.64 |
| BHZL8 | 27,840 | 14,235 | 13,605 | 787,038 | 28.27 |
| BHZL9 | 27,841 | 14,263 | 13,578 | 662,606 | 23.8 |
| **DD** |  |  |  |  |  |
| LNDD11 | 25,962 | 10,579 | 15,383 | 268,993 | 10.36 |
| LNDD12 | 28,781 | 14,900 | 13,881 | 798,785 | 27.75 |
| LNDD13 | 22,719 | 9,007 | 13,712 | 156,778 | 6.9 |
| LNDD15 | 28,196 | 14,775 | 13,421 | 717,871 | 25.46 |
| LNDD16 | 29,205 | 15,169 | 14,036 | 1,034,940 | 35.44 |
| LNDD17 | 29,072 | 15,029 | 14,043 | 800,111 | 27.52 |
| LNDD18 | 28,701 | 15,080 | 13,621 | 873,037 | 30.42 |
| LNDD19 | 29,181 | 15,159 | 14,022 | 996,857 | 34.16 |
| LNDD1 | 27,733 | 11,406 | 16,327 | 359,674 | 12.97 |
| LNDD20 | 27,972 | 14,512 | 13,460 | 851,115 | 30.43 |
| LNDD3 | 28,031 | 14,827 | 13,204 | 584,705 | 20.86 |
| LNDD5 | 28,818 | 15,017 | 13,801 | 1,013,356 | 35.16 |
| LNDD6 | 24,084 | 11,881 | 12,203 | 460,778 | 19.13 |
| LNDD7 | 28,180 | 14,764 | 13,416 | 723,499 | 25.67 |
| average | 27,799 | 13,769 | 14,030 | 644,304 | 22.99 |
