# Supplementary material for: Genome-Wide SNP Discovery, Genotyping and Their Preliminary Applications for Population Genetic Inference in Spotted Sea Bass (Lateolabrax maculatus)
Source: PLoS One. 2016 Jun 23;11(6):e0157809. doi: 10.1371/journal.pone.0157809 (PMC4919078; doi:10.1371/journal.pone.0157809)
Supplement: S3 Table — (DOCX) [file pone.0157809.s003.docx]

**S3** **Table. A list of the 55 best-quality BLASTx matches with E-value < 1E-6.**

| **Contig Name** | **Description** | **Length** | **Hits** | **min.** | **mean Similarity** |
| --- | --- | --- | --- | --- | --- |
|  |  |  |  | **e-value** |  |
| 2174006 | terminal uridylyltransferase 4 involved in ploy(A) RNA binding, RNA uridylytransferase activity and zinc ion binding. | 545 | 20 | 4.00E-21 | 100% |
| 2156905 | ubx domain-containing protein 6, which encodes a peripheral membrane protein that contains UBX domain. | 501 | 20 | 4.83E-12 | 99.40% |
| 136359 | golgi apparatus protein 1, which plays roles in negative regulation of protein processing and negative regulation of transforming growth factor beta receptor signaling pathway. | 518 | 20 | 8.27E-31 | 98.20% |
| 1733564 | probable e3 ubiquitin-protein ligase herc1, which is involved in membrane trafficking via some guanine nucleotide exchange factor (GEF) activity and its ability to bind clathrin. | 564 | 20 | 1.95E-39 | 97.25% |
| 91260 | agrin-like isoform x2 | 500 | 20 | 1.49E-54 | 92.85% |
| 2656317 | nucleolar protein 4-like | 441 | 20 | 7.13E-26 | 92.75% |
| 628717 | zinc finger protein 423-like isoform x3 | 580 | 20 | 1.58E-41 | 90.95% |
| 3052505 | aryl hydrocarbon receptor nuclear translocator-like protein 2 isoform x1 | 553 | 20 | 2.55E-14 | 90.25% |
| 1205767 | r-spondin-2- partial | 495 | 20 | 9.87E-17 | 89.90% |
| 2441721 | insulin-like growth factor-binding protein 3 | 497 | 20 | 8.30E-20 | 88.95% |
| 2645499 | protein fam50a | 368 | 20 | 2.00E-23 | 86.35% |
| 958860 | u6 snrna-associated sm-like protein lsm3 | 406 | 20 | 5.01E-20 | 85.25% |
| 432734 | cullin-associated nedd8-dissociated protein 1-like | 549 | 20 | 4.76E-26 | 84.60% |
| 2535893 | chromodomain-helicase-dna-binding protein 6-like | 527 | 20 | 4.75E-36 | 83.85% |
| 2861788 | protein nlrc3 | 573 | 20 | 3.51E-20 | 83.30% |
| 626205 | btb poz domain-containing protein 10-like isoform x1 | 632 | 20 | 7.39E-17 | 83.20% |
| 1450955 | rna-directed dna polymerase from mobile element jockey- partial | 626 | 20 | 1.08E-79 | 82.40% |
| 283613 | rna-directed dna polymerase from transposon bs | 586 | 20 | 1.93E-17 | 80.55% |
| 1075810 | golgi apparatus protein 1 | 570 | 20 | 3.47E-32 | 79.45% |
| 2722643 | spatacsin | 484 | 20 | 5.36E-33 | 79.30% |
| 3060068 | interferon-induced protein with tetratricopeptide repeats 5-like | 569 | 20 | 5.30E-17 | 78.90% |
| 427869 | stam-binding protein | 573 | 20 | 1.60E-17 | 77.35% |
| 1782285 | diacylglycerol kinase zeta isoform x1 | 520 | 20 | 3.33E-29 | 76.95% |
| 1751115 | ras-related protein rab-12 | 555 | 4 | 2.22E-11 | 73.50% |
| 369833 | atp-dependent rna helicase tdrd12 | 570 | 20 | 2.20E-14 | 73.25% |
| 690087 | kunitz-type protease inhibitor 1-like | 534 | 20 | 7.17E-17 | 72.40% |
| 2031533 | agouti-related | 570 | 20 | 4.39E-16 | 71.30% |
| 474018 | transcription factor | 522 | 2 | 4.02E-13 | 70.50% |
| 2208622 | titin-like isoform x1 | 584 | 19 | 5.68E-15 | 70.21% |
| 2005176 | nuclear valosin-containing | 600 | 20 | 1.70E-21 | 69.85% |
| 918013 | atp-binding cassette sub-family f member 3 | 546 | 15 | 2.15E-10 | 69.67% |
| 2312120 | reverse transcriptase-like protein | 461 | 20 | 2.90E-83 | 68.85% |
| 16835 | mitogen-activated protein kinase 14a-like | 476 | 20 | 4.79E-10 | 66.85% |
| 525464 | nucleic acid binding protein | 555 | 15 | 5.22E-16 | 65.07% |
| 1799965 | septin-6 isoform x3 | 585 | 20 | 4.89E-13 | 64.45% |
| 3027366_2 | PREDICTED: uncharacterized protein LOC103908834 | 266 | 20 | 1.52E-21 | 63.45% |
| 2220874 | mrg morf4l-binding protein | 534 | 20 | 2.57E-11 | 62.15% |
| 105161 | rna-directed dna polymerase from mobile element jockey-like | 546 | 20 | 2.32E-13 | 61.75% |
| 2305348 | ankyrin repeat domain-containing protein 27-like | 621 | 11 | 2.49E-07 | 61.73% |
| 1699444 | rna-directed dna polymerase from mobile element jockey | 578 | 20 | 9.55E-36 | 61.60% |
| 2602294 | death-associated protein kinase 1-like | 525 | 20 | 1.28E-07 | 58.25% |
| 3104866 | gastrula zinc finger | 466 | 20 | 1.25E-15 | 58.00% |
| 352269 | hba1_cotgo ame: full=hemoglobin subunit alpha-1 ame: full=alpha-1-globin ame: full=hemoglobin alpha-1 chain | 509 | 5 | 2.59E-10 | 56.80% |
| 2583004 | zinc finger protein zfpm2-like isoform x2 | 276 | 20 | 1.06E-13 | 56.15% |
| 432419 | zinc finger protein 648-like | 550 | 20 | 1.95E-71 | 55.90% |
| 1242038 | lipase maturation factor 2 | 523 | 20 | 9.29E-39 | 55.45% |
| 612117 | c-binding protein | 522 | 20 | 1.74E-41 | 54.55% |
| 283063 | snake venom metalloprotease inhibitor 02a10 | 521 | 20 | 5.94E-08 | 54.10% |
| 1650894 | plectin-like isoform x1 | 517 | 20 | 2.49E-14 | 54.10% |
| 840631 | ankyrin repeat domain-containing protein 27 | 575 | 20 | 2.69E-07 | 53.30% |
| 1161196 | regulator of g-protein signaling 12-like isoform x1 | 528 | 18 | 8.41E-06 | 52.39% |
| 437386 | pol-like protein | 569 | 20 | 1.13E-58 | 51.35% |
| 375059 | gag-pol polyprotein | 572 | 20 | 1.01E-07 | 51.30% |
| 1787491 | pleckstrin homology domain-containing family g member 7 isoform x2 | 592 | 7 | 8.16E-36 | 48.43% |
| 2238563 | protein fam50a | 563 | 20 | 7.19E-13 | 41.55% |
